# Hemichannels: new roles in astroglial function

**DOI:** 10.3389/fphys.2014.00193

**Published:** 2014-06-17

**Authors:** Juan A. Orellana, Jimmy Stehberg

**Affiliations:** ^1^Departamento de Neurología, Escuela de Medicina, Pontificia Universidad Católica de ChileSantiago, Chile; ^2^Laboratorio de Neurobiología, Centro de Investigaciones Médicas, Facultad de Ciencias Biológicas and Facultad de Medicina, Universidad Andrés BelloSantiago, Chile

**Keywords:** astrocytes, hemichannel, calcium waves, tripartite synapse, connexins, brain functions

## Abstract

The role of astrocytes in brain function has evolved over the last decade, from support cells to active participants in the neuronal synapse through the release of “gliotransmitters.”Astrocytes express receptors for most neurotransmitters and respond to them through Ca^2+^ intracellular oscillations and propagation of intercellular Ca^2+^ waves. While such waves are able to propagate among neighboring astrocytes through gap junctions, thereby activating several astrocytes simultaneously, they can also trigger the release of gliotransmitters, including glutamate, d-serine, glycine, ATP, adenosine, or GABA. There are several mechanisms by which gliotransmitter release occurs, including functional hemichannels. These gliotransmitters can activate neighboring astrocytes and participate in the propagation of intercellular Ca^2+^ waves, or activate pre- and post-synaptic receptors, including NMDA, AMPA, and purinergic receptors. In consequence, hemichannels could play a pivotal role in astrocyte-to-astrocyte communication and astrocyte-to-neuron cross-talk. Recent evidence suggests that astroglial hemichannels are involved in higher brain functions including memory and glucose sensing. The present review will focus on the role of hemichannels in astrocyte-to-astrocyte and astrocyte-to neuron communication and in brain physiology.

## Astrocytes: general background

Astrocytes are spongiform-shaped glial cells (Bushong et al., 2002 and Ogata and Kosaka, 2002) that, contrary to common belief, are the most abundant cell type in the brain. They are divided into two major types based on their morphology, biochemistry, development, and location within the central nervous system (CNS): protoplasmic and fibrous (Miller and Raff, 1984). Given their numerous functions, several studies have tried to differentiate subpopulations of astrocytes (Lerea and McCarthy, 1989). However, such attempts have been unsuccessful due to the extraordinary capacity of astrocytes to adapt to their surrounding environment by changing the expression of a vast number of proteins. This is particularly evident in primary cultures, where they show fast changes in the expression of several receptors for neuro- and gliotransmitters (Shao and McCarthy, 1993; Shao et al., 1994). There is a remarkable heterogeneity in astrocyte populations, between different species, brain regions and within a brain region, in terms of their receptor expression, gap junction coupling, membrane currents, and their morphology (Matyash and Kettenmann, 2010; Zhang and Barres, 2010; Theis and Giaume, 2012).

Astrocytes have pivotal roles in brain function, including the maintenance of osmotic balance and optimal ionic conditions for neurons (Kimelberg, 2005), K^+^ clearance from the extracellular space (Wallraff et al., 2006; Sibille et al., 2013), glucose and lactate metabolism (Allaman et al., 2011), neurotransmitter recycling of the two most abundant neurotransmitters in the brain, glutamate and GABA (Simard and Nedergaard, 2004), and immune responses (Dong and Benveniste, 2001; Farina et al., 2007). Moreover, astrocytes have end-feet that cover blood vessels and release vasoactive substances to regulate cerebral microcirculation (Anderson and Nedergaard, 2003; Zonta et al., 2003; Takano et al., 2006) and blood brain barrier (BBB) permeability (Alvarez et al., 2013). In fact, their end-feet physically constitute part of the BBB. Finally, astrocytes communicate with neurons through transmitters which are released into neighboring synapses, now called “gliotransmitters.” It is not within the scope of the present review to comment on the above functions, for which we have cited very comprehensive reviews, which are highly recommended. The present review will focus on the possible role of hemichannels in astroglial function and brain physiology.

## Astrocytes respond to synaptic neurotransmitters

Astrocytes express membrane receptors for almost all major neurotransmitters and neuromodulators, and possess ion channels and intracellular signaling cascades that allow them to respond within milliseconds to neuronal activity and neurotransmitters released at synapses. These fast responses occur mainly as changes in intracellular free Ca^2+^ concentration ([Ca^2+^]_i_) (MacVicar and Tse, 1988; Marrero et al., 1989; Usowic et al., 1989; Barres et al., 1990; Salm and McCarthy, 1990; McCarthy and Salm, 1991). The mechanism by which astroglial activation occurs is believed to start when neurotransmitters released from neurons at the synapse activate receptors at the astroglial membrane, inducing activation of phospholipase C (PLC) and the concomitant production of IP_3_. The latter then triggers the release of intracellular Ca^2+^ stored at the endoplasmatic reticulum (Sheppard et al., 1997; Golovina and Blaustein, 2000; Scemes, 2000), which opens hemichannels (De Vuyst et al., 2009) and activates other Ca^2+^ dependent gliotransmitter release mechanisms including exocytosis. Hemichannels are hexameric plasma membrane channels formed by two different families of membrane proteins: connexins (Cx) and pannexins (Panx). Although these proteins do not share a relevant homologous primary structure, they have similar secondary and tertiary structures with four α-helical transmembrane domains, connected by one cytoplasmic and two extracellular loops, and intracellular N- and C-termini. Importantly, hemichannel opening allows the release of glutamate (Ye et al., 2003; Takeuchi et al., 2006; Kang et al., 2008; Jiang et al., 2011; Orellana et al., 2011a,b), ATP (Stout et al., 2002; Iglesias et al., 2009; Orellana et al., 2011a,b; Torres et al., 2012) and other gliotransmitters into the extracellular space. Given that astrocytes express NMDA receptors insensitive to blocking by extracellular Mg^2+^, and are activated following physiological synaptic transmission (Verkhratsky and Kirchhoff, 2007) and through purinergic receptor channels (Idestrup and Salter, 1998; Zhu and Kimelberg, 2004; Lalo et al., 2008; Illes et al., 2012), ATP and glutamate released via hemichannels onto the extracellular space can activate purinergic or NMDA receptor channels located in the same astrocyte or in neighboring astrocytes, inducing changes in [Ca^2+^]_i_ (Zanotti and Charles, 1997; Guthrie et al., 1999). Moreover, because astrocytes envelope synapses, the release of glutamate, ATP, and other gliotransmitters also activates neighboring pre- and post-synaptic neurons, modulating synaptic activity (Dani et al., 1992; Nedergaard, 1994; Parpura et al., 1994; Kang et al., 1998; Parri et al., 2001). In fact, astrocytes stimulated by amyloid β-peptide (Aβ) release ATP and glutamate via connexin 43 (Cx43) hemichannels (Orellana et al., 2011a). Importantly, both of these gliotransmitters released by astrocytes have been shown to increase Panx1 hemichannel activity in neurons by activating P2X7 and NMDA receptors, resulting in further neuronal death. Given that high [Ca^2+^]_i_ enhances Panx1 hemichannel activity (Locovei et al., 2006), it is likely that purinergic and glutamatergic receptor activation leads to Panx1 hemichannel opening by inducing Ca^2+^ influx or by releasing Ca^2+^ from intracellular stores via activation of IP_3_ receptors (Zanotti and Charles, 1997; Guthrie et al., 1999; Stout et al., 2002; Suadicani et al., 2006).

As reported by Cornell-Bell et al. (1990), both the initial increase, and sustained oscillation of [Ca^2+^]_i_ induced by glutamate in astrocytes, depend on the concentration of the latter. Indeed, under low glutamate concentrations (>1 μM), [Ca^2+^]_i_ oscillations in single astrocytes appear locally, asynchronously and are short-lasting, whereas concentrations above 100 μM generate astrocyte-to-astrocyte propagating Ca^2+^ waves which last up to 30 min (Cornell-Bell et al., 1990). These intercellular Ca^2+^ waves can be propagated among adjacent astrocytes through Cx43 and Cx30 gap junction channels (GJCs) (Cornell-Bell et al., 1990; Charles et al., 1991; Enkvist and McCarthy, 1992; Finkbeiner, 1992; Venance et al., 1995; Leybaert et al., 1998; Scemes et al., 1998; Blomstrand et al., 1999; Suadicani et al., 2006) or by the Ca^2+^-dependent release of ATP and glutamate and further activation of purinergic or glutamate receptors in neighboring astrocytes (Zanotti and Charles, 1997; Guthrie et al., 1999; reviewed in Bennett et al., 2003; Leybaert and Sanderson, 2012). GJCs are intercellular channels formed by docking of two hemichannels, one provided by each adjacent cell (Sáez et al., 2003). These channels connect the cytoplasmic compartments of adjacent cells, favoring the intercellular exchange of metabolites (e.g., ADP, ATP, glucose and glutathione), second messengers (e.g., cAMP and IP_3_) and ions (e.g., Ca^2+^, K^+^ and Na^+^).

To obtain a Ca^2+^ wave, the released Ca^2+^ needs to be significantly amplified. This amplification is mediated at least in part by the capacity of Ca^2+^ itself to activate both IP_3_ receptors (Finch and Turner, 1991; Bezprozvanny and Ehrlich, 1995) and phospholipase C (Berridge, 1993; Venance et al., 1997) as well as through other mechanisms reviewed in Leybaert and Sanderson, 2012. It has been reported that such Ca^2+^ waves originate from a localized area of the cell (Shao et al., 1994) and spread throughout the cell and into other cells in a non-decremented manner (Shao et al., 1994). Moreover, it has been suggested that astroglial Ca^2+^ responses occur once a “threshold” is reached and in an “all-or-none” manner reminiscent of neuronal action potentials (Shao and McCarthy, 1993; Shao et al., 1994). In consequence, the formation of Ca^2+^ waves is intriguing, as it must include a mechanism that will set this threshold, which may depend on the isoform of the IP_3_ receptor and on the concentration of IP_3_ and Ca^2+^. In neurons, during the generation of an action potential this threshold is defined by the membrane potential required to activate voltage-dependent Na^+^ channels, which are densely located at the axon hillock and along the axon. The mechanism by which Ca^2+^ oscillations or fluctuations are integrated into a threshold that determines the triggering of a Ca^2+^ wave remains unclear, although some hypotheses have been postulated (see Leybaert and Sanderson, 2012). Nonetheless, the idea of a Ca^2+^ wave being a distinct phenomenon rather than just the consequence of a larger increase in [Ca^2+^]_i_ is also supported by other studies. In a study by McCarthy and Salm (1991), primary astrocytes were exposed to different neurotransmitter agonists and showed different Ca^2+^ responses to different neurotransmitters in distinct subpopulations. Interestingly, they found that astrocytes respond to neurotransmitter agonists by either a Ca^2+^ wave or [Ca^2+^]_i_ oscillations (McCarthy and Salm, 1991), that is, if a cell population responded to a given agonist with a Ca^2+^ wave, it may respond to another agonist with [Ca^2+^]_i_ oscillations and vice versa (McCarthy and Salm, 1991). This suggests, that in a manner similar to neuronal summation of post-synaptic evoked potentials, fluctuations in [Ca^2+^]_i_ may be integrated additively to generate propagating Ca^2+^ waves that can activate entire astroglial networks.

In cultured astrocytes, [Ca^2+^]_i_ oscillations can occur spontaneously in the absence of neuronal activation (Aguado et al., 2002; Perea and Araque, 2005), but can be regulated by neuronal activation and transmitter release. Ca^2+^ waves, on the other hand, appear in response to neurotransmitters, but given that astrocytes so far have been studied *in vitro*, it has been argued that Ca^2+^ waves may appear only in non-physiological conditions or in pathology (Scemes and Giaume, 2006). *In vivo* it is difficult to differentiate Ca^2+^ waves from [Ca^2+^]_i_ oscillations due to technical difficulties. However, [Ca^2+^]_i_ oscillations have been observed *in vivo* using imaging techniques under physiological conditions. These [Ca^2+^]_i_ oscillations in astrocytes were found to be correlated to neuronal discharges (Hirase et al., 2004), and appear in response to sensory stimulation (Cirillo et al., 2012; Lind et al., 2013), electrical stimulation of afferent fibers (Johannssen and Helmchen, 2010) or ATP (Ding, 2012) and at speeds sufficiently fast to occur concomitantly with neuronal activity and hemodynamic changes (Lind et al., 2013). A very recent study has reported that whisker stimulation in awake, behaving mice induces very large Ca^2+^ astroglial responses spread over a large portion of cortex and which are modulated by subcortical noradrenergic input, but not by intracortical glutamate (Ding et al., 2013).

## Functional hemichannels in astrocytes

Although the principal connexin in astrocytes is Cx43 (Dermietzel et al., 1989), they also express Cx30 GJCs (Nagy et al., 1999) and Pannexin 1 (Panx1; Iglesias et al., 2009) and Panx2 (Zappalá et al., 2007). Some studies, however, have also reported low levels of Cx26, Cx40, and Cx45 (Dermietzel et al., 1989, 2000; Nagy et al., 1997, 1999). Yet, despite the observations of these latter studies, astrocytes from Cx43/Cx30 double knockout mice fail to show gap junction-mediated communication (Wallraff et al., 2006; Rouach et al., 2008) indicating that Cx43 and Cx30 are the main functional connexins in astrocytes.

Cx43 hemichannels have mostly been studied *in vitro* using transfected and primary cells, as well as from acute slice experiments (Ye et al., 2003; Orellana et al., 2011a; Chen et al., 2012; Torres et al., 2012). The conditions found *in vitro* that favor Cx43 hemichannel opening seemed non-physiological at first, leading to a debate on its functionality under physiological conditions. This stems from an earlier belief that hemichannels opened at only highly depolarized membrane potentials (around 60 mV), making their opening virtually impossible in non-excitable cells like astrocytes, which show no large changes in membrane potential. However, recent studies have shown hemichannel opening also at negative membrane potentials (Retamal et al., 2007; Orellana et al., 2011a,b). Indeed, hemichannel-mediated uptake of several dyes (e.g., ethidium, propidium, TOPRO, YOPRO) occurs at resting membrane potentials (Contreras et al., 2003), suggesting that hemichannel opening may also be present at resting membrane conditions.

High levels of intracellular [Ca^2+^]_i_ and low extracellular Ca^2+^ ([Ca^2+^]_o_) increase opening probability of Cx43 hemichannels (Stout and Charles, 2003; Bao et al., 2004; Wang et al., 2012) whereas normal extracellular [Ca^2+^]_o_ closes them (Stout and Charles, 2003). Cx43 hemichannels have been reported to mediate the release of gliotransmitters (glutamate, ATP, glutathione) from astrocytes and glioma cells (Stout et al., 2002; Ye et al., 2003). Ye et al. (2003) demonstrated that low extracellular [Ca^2+^]_o_ induces glutamate release from astrocytes through Cx43 hemichannels in an exocytosis-independent manner and involves neither large pore anion channels, purinergic receptors, nor reversal of the glutamate transporter (Ye et al., 2003). This idea was further supported by reports showing ATP release from glioma cells overexpressing Cx43 and exposed to zero extracellular [Ca^2+^]_o_ (Ye et al., 2003; Contreras et al., 2004; Retamal et al., 2006). Other studies, however, have reported ATP release from astrocytes also mediated by the P2X7 receptor, Panx1 hemichannels, and exocytosis (Parpura et al., 1994; Coco et al., 2003; Bezzi et al., 2004; Mothet et al., 2005; Pascual et al., 2005; Garré et al., 2010). This suggests that ATP is released by astrocytes through different mechanisms. In a study by Garré et al. (2010), it was reported that pharmacological blockade of vesicles inhibited only early ATP release from astrocytes, while later release was reported to be mediated by P2X7 receptor activation as well by Panx1 and Cx43 hemichannel opening, suggesting that each release mechanism may occur at different periods.

## Role of astroglial connexin and pannexin hemichannels in gliotransmitter release at the synapse

Astrocytes release gliotransmitters into neuronal synapses, giving rise to what is now known as the tripartite synapse (Araque et al., 1998), implying a synapse between a pre- and post-synaptic neuron and their bidirectional communication with one astrocyte. Glutamate is the most important and abundant excitatory neurotransmitter of the CNS and one the most ubiquitous gliotransmitters released by astrocytes (Navarrete et al., 2012). Multiple mechanisms have been proposed to explain the release of glutamate from astrocytes, including hemichannels (Ye et al., 2003), anion channels (Wang et al., 2013), and exocytosis (Parpura et al., 1994; Coco et al., 2003; Bezzi et al., 2004; Mothet et al., 2005; Pascual et al., 2005 but see Wang et al., 2013). The other major neurotransmitter is GABA, the principal inhibitory neurotransmitter of the CNS. Although GABA is abundantly released by interneurons, it is also released by astrocytes (Lee et al., 2011).

Perhaps some of the best known gliotransmitters are D-serine and glycine, which are required for NMDAR activation of post-synaptic neurons and necessary for glutamate-mediated synaptic plasticity (Panatier et al., 2006; Henneberger et al., 2010; Hogerton and Bowser, 2013; Kang et al., 2013). D-serine has been reported to be released from astrocytes via large vesicles (Kang et al., 2013) and exocytosis (Parpura et al., 1994; Coco et al., 2003; Bezzi et al., 2004; Mothet et al., 2005; Pascual et al., 2005). It must be noted that a recent report has suggested that neurons may also release D-serine and glycine (Balu et al., 2014 and Ehmsen et al., 2013) both involved in regulating synaptic plasticity (Rosenberg et al., 2013). Until now, there has been no evidence indicating that astroglial hemichannels can release either D-serine or glycine.

As stated earlier, ATP both activates astrocytes and is also released by them. Additionally, it appears to suppress glutamatergic synapses (Zhang et al., 2003; Cao et al., 2013a) and can be turned into adenosine, which decreases excitatory transmission (Dunwiddie and Diao, 1994; Dunwiddie et al., 1997, but see Fujita et al., 2012). ATP has been shown to be released through hemichannels (Stout et al., 2002; Kang et al., 2008), P2X7 channels (Suadicani et al., 2006) and exocytosis (Parpura et al., 1994; Coco et al., 2003; Bezzi et al., 2004; Mothet et al., 2005; Pascual et al., 2005). Another gliotransmitter, glutathione, is released in response to extracellular glutamate (Frade et al., 2008) through connexin hemichannels (Rana and Dringen, 2007). Other well-known gliotransmitters include BDNF (Parpura and Zorec, 2010) and taurine (Choe et al., 2012). Although, previous studies have shown that taurine could be released via astroglial hemichannels (Stridh 2006/2008), further studies are necessary to elucidate whether BDNF could be released by the same pathway.

## Astrocytic hemichannels in brain function

**Figure 1:**
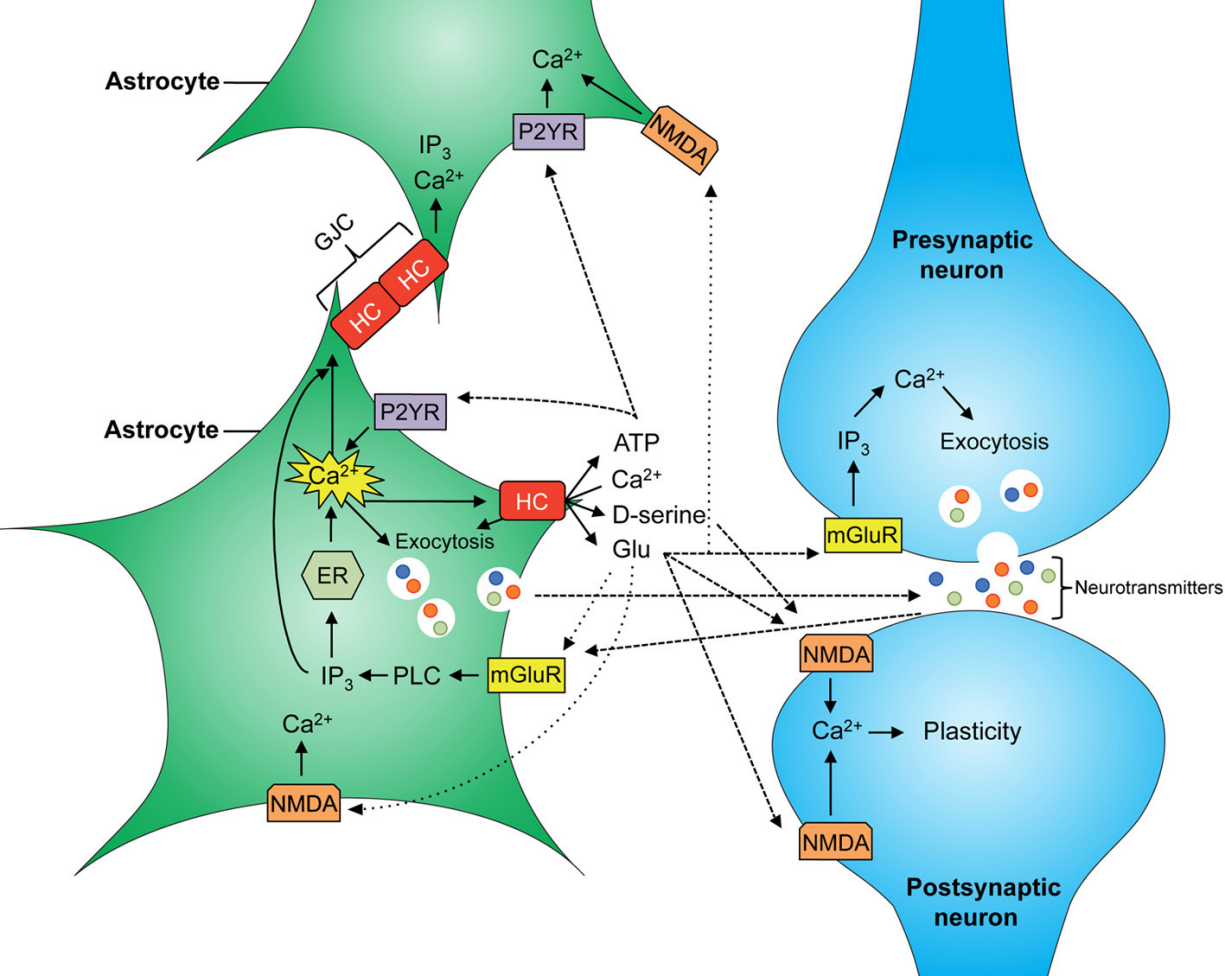
**Scheme of major astrocyte signaling associated to gliotransmitter release**. Increased intracellular free Ca^2+^ concentration ([Ca^2+^])_i_ allows the release of gliotransmitters into the synaptic cleft through vesicles and hemichannels (HCs). D-serine, glycine, and glutamate released by astrocytes can activate NMDA receptors at the post-synaptic neuron and modulate neuronal plasticity. Astroglial glutamate also binds to mGluR at the presynaptic neuron increasing neuronal release of glutamate into the synapse. In astrocytes, increasing ([Ca^2+^])_i_ allows Ca^2+^ wave propagation between astrocytes, mediated by gap junction channels (GJCs) and by release of glutamate and ATP, resulting in further activation of NMDA and P2YR receptors at neighboring astrocytes, respectively.

Given that astrocytes participate in the tripartite synapse, their contribution to brain function is as wide as that of neurons; taking into account their other functions (microcirculation, BBB formation), perhaps even more so (for a schematic of main astrocytic signaling cascades see Figure 1). Hemichannels contribute to the release of glutamate which is necessary for NMDAR-dependent synaptic plasticity (Henneberger et al., 2010; Navarrete et al., 2012). In fact, recently it was shown that blockade of Cx43 hemichannels in the basolateral amygdala by microinjection of mimetic peptides impairs memory consolidation but not short-term memory (Stehberg et al., 2012). Given that this study was performed using rodent fear conditioning, which is the most accepted model of post-traumatic stress disorder in animals, it would be plausible to suggest that Cx43 hemichannels may have a role in the establishment of memories in general and traumatic memories in particular.

## Potential role of astroglial connexin and pannexin hemichannels in psychiatric diseases

There is to-date no direct evidence linking astrocytic hemichannels and psychiatric disorders, so one can only speculate what their role might be. Studies have shown abnormal expression of glial fibrillary acid protein (GFAP)—a marker for astrocytes—in the post-mortem brain of patients with major depression (Bowley et al., 2002; Altshuler et al., 2010; Rajkowska and Stockmeier, 2013), while other studies have shown reduced density of astrocytes from clinical (Ongur et al., 1998; Cotter et al., 2001; Bremner et al., 2002), post-mortem (Cotter et al., 2001), and preclinical (Banasr and Duman, 2008; Banasr et al., 2010) studies, suggesting that the density and reactivity of astrocytes are reduced in this mood disorder.

Moreover, accumulating evidence suggests that antidepressants act on astrocytes (for reviews see Czéh and Di Benedetto, 2013; Etiévant et al., 2013). These express a variety of receptors including monoaminergic transporters and receptors, leading to the possibility that antidepressants exert their effects at least in part through modifying astroglial function (Peng and Huang, 2012; Quesseveur et al., 2013a). In this sense, it's been demonstrated that application of antidepressants on rodent primary astrocyte cultures may elicit Ca^2+^ waves, Ca^2+^ oscillations, release of gliotransmitters, glucose metabolites, and neurotrophic factors (Hisaoka et al., 2011), whereas studies in post-mortem human brain tissue suggest that antidepressants may reverse major depression associated glial reductions in the amygdala (Bowley et al., 2002). Interestingly, many transmitters released by astrocytes have antidepressant or anxiolytic effects. To this effect, acute D-serine treatment (800–2700 mg/Kg) produces antidepressant-like effects in rodents (Malkesman et al., 2012), astroglial release of ATP has been shown to modulate depressive-like behaviors (Cao et al., 2013b), GABA agonists are well known to have anxiolytic effects (the mechanism of action for benzodiazepines), while overexpression of astrocytic BDNF produces anxiolytic effects (Quesseveur et al., 2013b). Given that the release of all the above could be mediated at least in part by hemichannels, it is probable that mood depends on hemichannel activity.

Studies have also shown abnormal expression of GFAP in the post-mortem brain of patients with schizophrenia (Toro et al., 2006; Feresten et al., 2013). In fact, Khan and colleagues (Khan et al., 2001) found by electron microscopy that roughly 1/3 of D2 dopamine receptors in the cortex are expressed in astrocytes, and that D2 receptor agonist quinpirole increases astroglial intracellular [Ca^2+^]_i_, suggesting that astrocytes may be a target for antipsychotics.

Finally, current evidence also suggests that astrocytes could be involved in drug abuse (Miguel-Hidalgo, 2009). All in all, given the pivotal role astrocytes play in brain function, and their active release of gliotransmitters into synapses, it is highly probable that they will become a target in the treatment of psychiatric diseases. In this respect, hemichannels constitute an attractive candidate for such treatment as they mediate gliotransmitter release at the synapse of glutamate, activating NMDA, and non NMDA-dependent mechanisms critical for synaptic plasticity and the release of ATP and adenosine which may decrease neuronal network excitation. Moreover, antidepressants and antipsychotics may act, at least in part, through various astroglial monoamine receptors and transporters to modulate cytoplasmic Ca^2+^ that controls hemichannel activity.

### Conflict of interest statement

The authors declare that the research was conducted in the absence of any commercial or financial relationships that could be construed as a potential conflict of interest.
